# Supplementary material for: The Nuclease Domain of E. coli RecBCD Helicase Regulates DNA Binding and Base Pair Melting
Source: J Mol Biol. Author manuscript; Available in PMC 2026 Apr 22. (PMC13101046; doi:10.1016/j.jmb.2025.169571)

**Supplementary Information**

**Supplementary Table 2**. Equilibrium constants of RecBCD (K_BCD_), RecB^∆Nuc^CD (K_B∆NucCD_) binding to 3’-dT_n_ or 5’-dT_n_ DNA ends in Buffer M275-10, 25°C, from fluorescence titrations.

| **Substrates** | **K_B∆NucCD_ (10^8^M^-1^)** | **K_BCD_ (10^8^M^-1^)** |
| --- | --- | --- |
| Blunt Ended | 0.47±0.04 | 0.14±0.05 |
| 3'-dT_2_ | 0.96±0.06 | 0.21±0.06 |
| 3'-dT_4_ | 3.3±0.2 | 0.47±0.02 |
| 3'-dT_6_ | 9.5±0.5 | 1.1±0.1 |
| 3'-dT_8_ | 7.2±0.3 |  |
| 3'-dT_10_ | 5.9±0.6 | 0.5±0.1 |
| 3'-dT_15_ | 0.6±0.1 | 0.1±0.01 |
|  |  |  |
| 5'-dT_6_ | 1.4±0.1 | 1.1±0.1 |
| 5'-dT_8_ | 2.3±0.1 |  |
| 5'-dT_10_ | 6.4±0.6 | 2.2±0.4 |
| 5'-dT_15_ | 6.5±0.7 | 2.3±0.2 |

**Supplementary Table 3.** Results of ITC experiments of RecB^ΔNuc^CD

| Substrate | Buffer M275-10 | | Cryo-EM Buffer | |
| --- | --- | --- | --- | --- |
|  | ΔH_obs_ (kcal/mol) | K (M^-1^) | ΔH_obs_(kcal/mol) | K (M^-1^) |
| blunt | +10.4±0.4 | 1.2(±0.4)E7 | +7.5±0.3 | >1E9 |
| dT_4_dT_4_ | -6.0±0.7 | >1E9 | -- | -- |
| dT_6_dT_6_ | -20±1 | >1E9 | -- | -- |
| dT_10_dT_10_ | -38±1 | >1E9 | -- | -- |
| dT_15_dT_15_ | -53±2 | >1E9 | -- | -- |
| dT_20_dT_20_ | -49±3 | >1E9 | -58±2 | >1E9 |
| dT_25_dT_25_ | -50±2 | >1E9 | -- | -- |
| dT_30_dT_30_ | -52±2 | 6.8(±0.8)E8 | -56±3 | >1E9 |

**Supplementary Table 4**. Local RMSD (Å) between regions of various structures

|  | BCD Class 1 vs BCD-DNA Class 1 | BCD Class 2 vs BCD-DNA Class 1 | BCD Class 3 vs BCD-DNA Class 1 | B^∆Nuc^CD vs BCD-DNA Class 1 | BCD Class 2 vs BCD Class 1 | BCD Class 3 vs BCD Class 1 | B^∆Nuc^CD vs BCD-DNA Class 2 | B^∆Nuc^CD-DNA vs BCD Class 1 | BCD-DNA Class1 vs BCD-DNA Class 2 |
| --- | --- | --- | --- | --- | --- | --- | --- | --- | --- |
| **RecB^2B^** | **3.6** | **4.9** | **2.3** | **4.1** | **4.7** | **3.4** | **3.5** | **3.6** | **1.8** |
| **RecC^Cter^** | **4.4** | **4.1** | **2.1** | **3.1** | **6.4** | **5.0** | **3.1** | **4.8** | **1.3** |
| RecB^2A^ | 2.4 | 3.4 | 3.5 | 2.3 | 3.0 | 3.3 | 1.8 | 3.6 | 2.5 |
| RecB^1A^ | 1.4 | 1.8 | 1.7 | 1.1 | 1.4 | 1.6 | 1.2 | 1.5 | 1.2 |
| RecC^Nter^ | 2.3 | 1.9 | 1.7 | 1.4 | 5.1 | 4.6 | 3.1 | 4.4 | 1.3 |

*In this table, the following structures are used for comparisons:

RecB^∆Nuc^CD – 8UNA

RecB^∆Nuc^CD-DNA – 8UNB

RecBCD class 1 – 7MR0

RecBCD class 2 – 7MR1

RecBCD class 3 – 7MR2

RecBCD-DNA class 1- 7MR3

RecBCD-DNA class 2 – 7MR4

**Supplementary Table 5.** Cryo-EM statistics for RecB^ΔNuc^CD and RecB^ΔNuc^CD-DNA structures

| **Models** | **B**^ΔNuc^**CD** | **B**^ΔNuc^**CD-DNA** |
| --- | --- | --- |
| **PDB Accession Code** | **8UNA** | **8UNB** |
| **Model Composition** |  |  |
| Nonhydrogen Atoms | 16387 | 17075 |
| Protein Residues | 2036 | 2038 |
| Nucleotides | 0 | 34 |
| **Bonds (RMSD)** |  |  |
| Length (Å) | 0.004 | 0.002 |
| Angle (°) | 0.82 | 0.48 |
| **Validation** |  |  |
| MoProbity Score | 1.54 | 1.63 |
| Clash Score | 4.83 | 7.17 |
| Rotamer outliers (%) | 0.17 | 0 |
| Cβ outliers (%) | 0 | 0 |
| CaBLAM outliers (%) | 2.26 | 2.23 |
| **Ramachandran plot (%)** |  |  |
| Outliers | 0.05 | 0.05 |
| Allowed | 4.16 | 3.5 |
| Favored | 95.79 | 96.45 |
| **B-factors (Å^2^)** |  |  |
| Protein | 56.79 | 66.17 |
| Nucleotides |  | 176.59 |


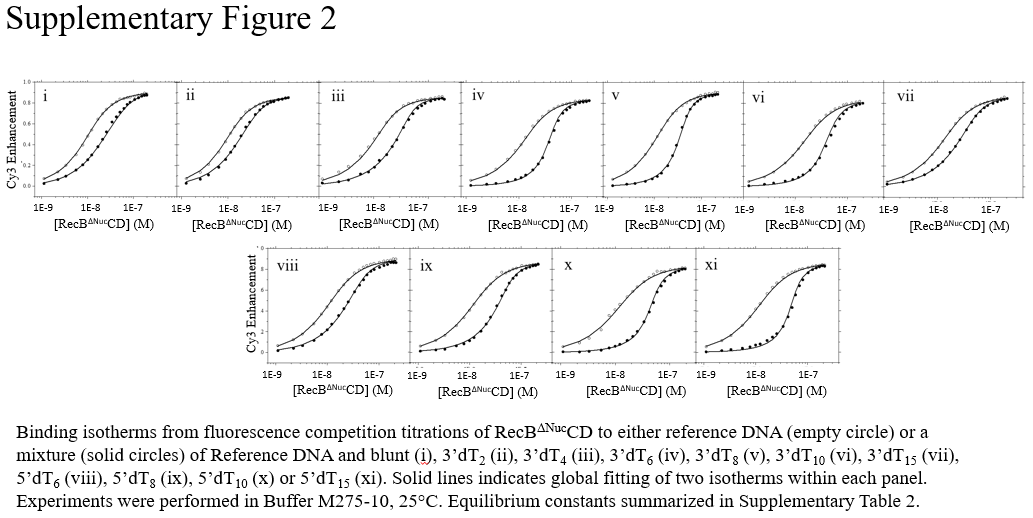


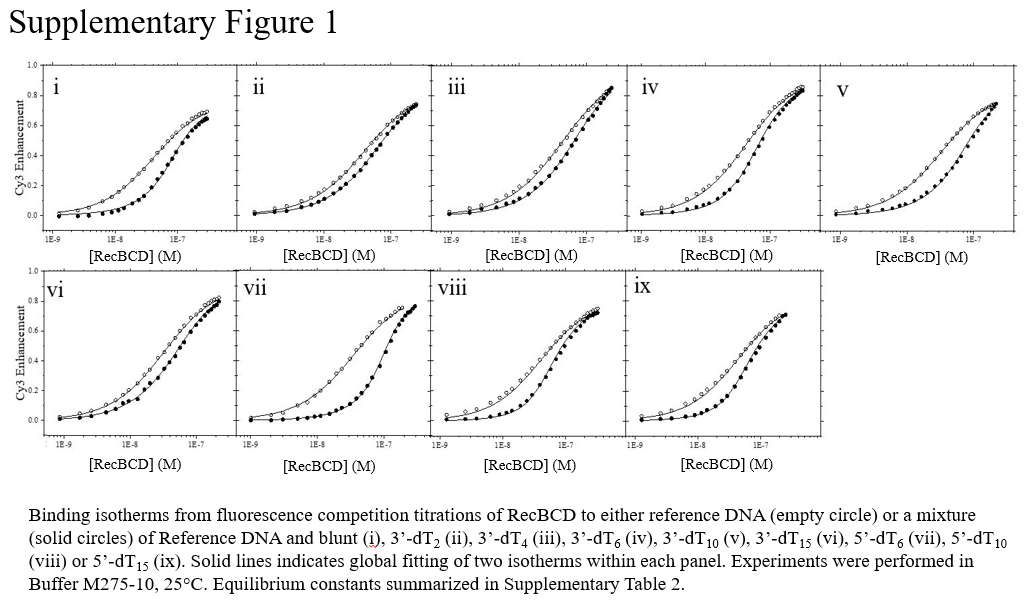

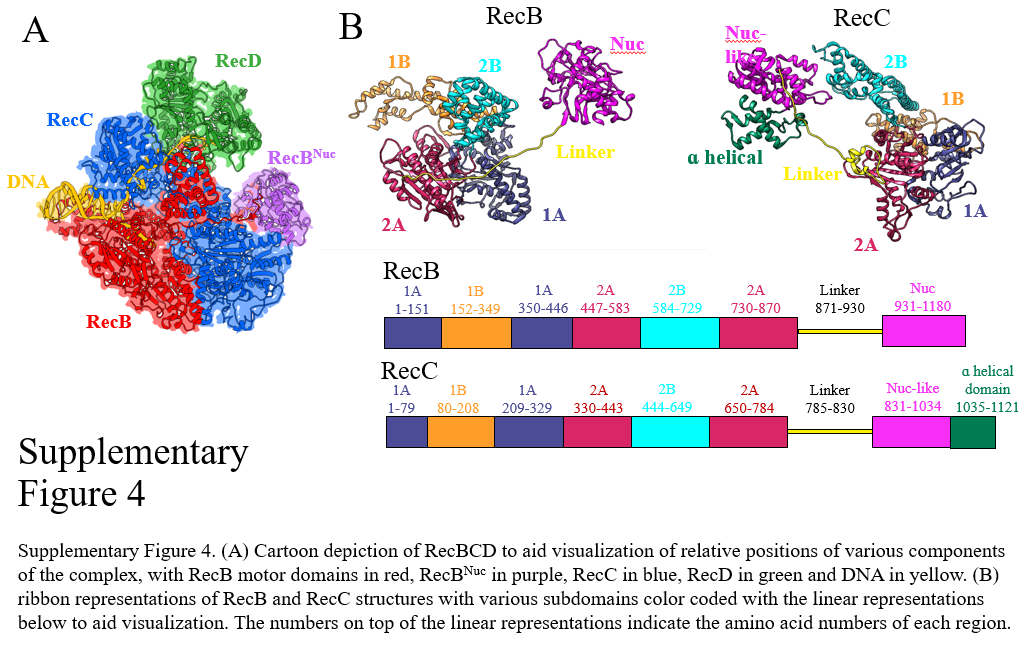

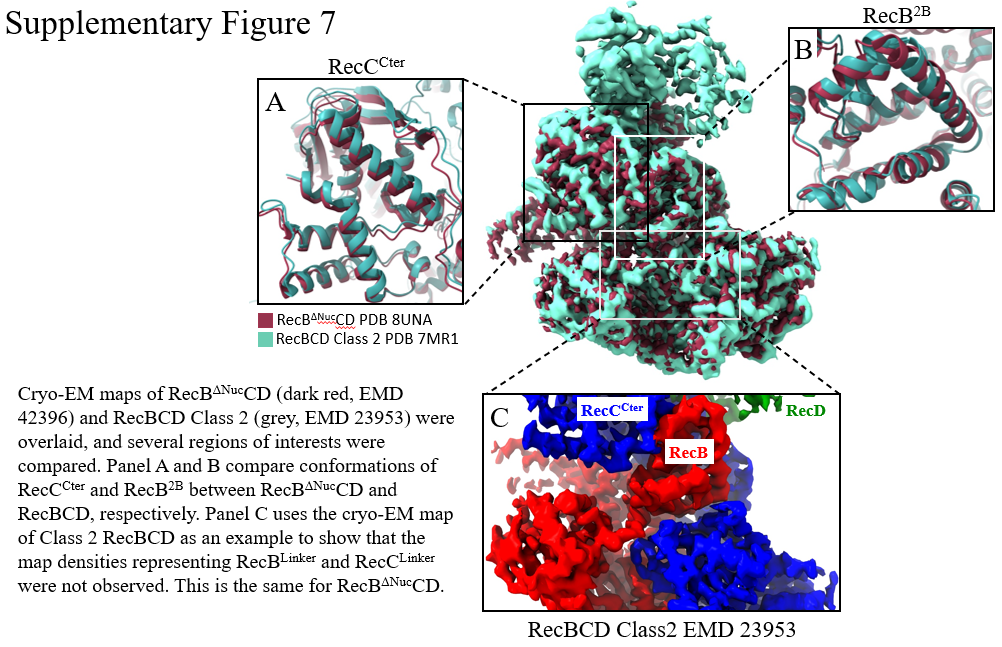

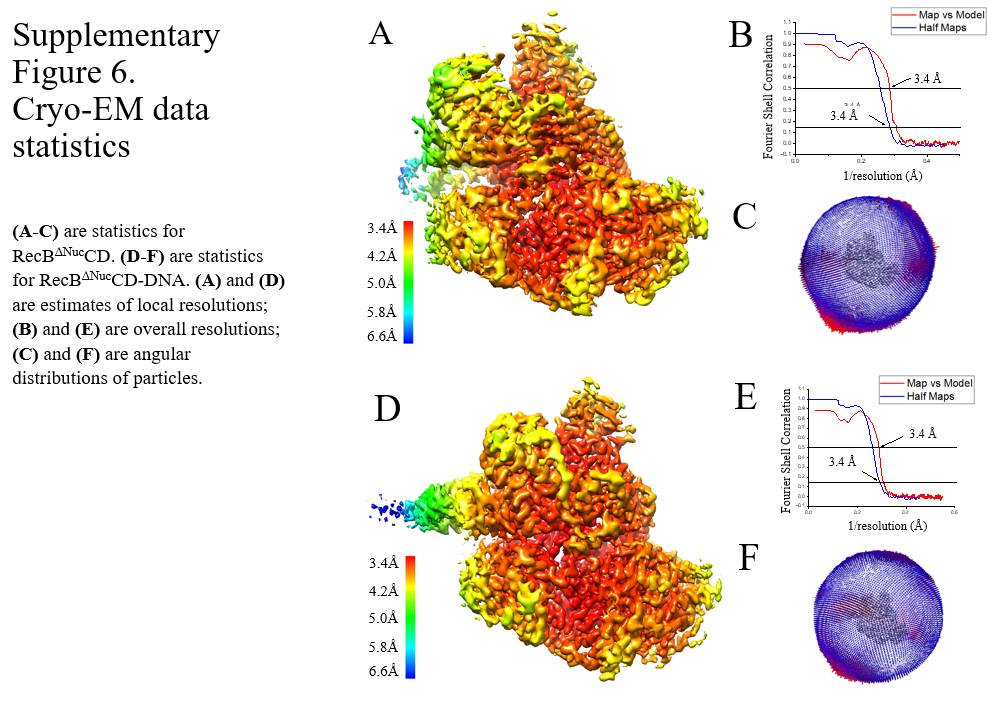

Supplement: 1 [file NIHMS2163069-supplement-1.docx]
